# Notes and Notices

**Published:** 1882-11

**Authors:** 


### NOTES AND NOTICES.

The New York Ophthalmic Hospital for Eye and Ear, corner Third Avenue and Twenty-third Street. Report for the month ending Sept. 30th, 1882:

Number of prescriptions, 3,505; number of new patients, 760; number of patients resident in the hospital, 14; average daily attendance, 135; largest daily attendance, 180.

### ET TU, BRUTE!

Infinitesimal doses are absolutely inoperative, and all so-called cures by them must be regarded as accidental or fortunate recoveries. Those homœopaths who use tangible doses must be regarded as fractions or unreasonable allopaths. Those who use the remedies and doses of the regular school may be looked upon as erring brethren who have placed themselves in such a false position that they can only be rescued by great kindness and consideration on the part of their more numerous and powerful fellow-practitioners.

All the great modern advances in therapeutics have been made in the regular school, which must be regarded as the newest, most progressive, and by far the most scientific one. Homœopathy has made no advances in its own direction during the last twenty-five years, and must be regarded as an old, almost effete, and dying system, which would long ago have ceased to exist as a school if its adherents had not adopted so much from the regular school.—*John C. Peters, M. D., Medical Record, Aug. 5th.*

